# Effectiveness of deltamethrin-impregnated dog collars on the incidence of canine infection by *Leishmania infantum*: A large scale intervention study in an endemic area in Brazil

**DOI:** 10.1371/journal.pone.0208613

**Published:** 2018-12-10

**Authors:** Wendel Coura-Vital, Gleisiane Gomes de Almeida Leal, Luana Araújo Marques, Aimara da Costa Pinheiro, Mariângela Carneiro, Alexandre Barbosa Reis

**Affiliations:** 1 Programa de Pós-Graduação em Ciências Farmacêuticas, Departamento de Análises Clínicas, Escola de Farmácia, Universidade Federal de Ouro Preto, Minas Gerais, Brazil; 2 Laboratório de Imunopatologia, Programa de Pós-Graduação em Ciências Biológicas, Núcleo de Pesquisa em Ciências Biológicas, Universidade Federal de Ouro Preto, Ouro Preto, Minas Gerais, Brazil; 3 Centro de Controle de Zoonoses, Secretaria Municipal de Saúde, Governador Valadares, Minas Gerais, Brazil; 4 Laboratório de Epidemiologia das Doenças Infecciosas e Parasitárias, Departamento de Parasitologia, Universidade Federal de Minas Gerais, Belo Horizonte, Minas Gerais, Brazil; 5 Instituto Nacional de Ciência e Tecnologia em Doenças Tropicais—INCT-DT, Salvador, Bahia, Brazil; Justus Liebig Universitat Giessen, GERMANY

## Abstract

To reduce morbidity and mortality caused by visceral leishmaniasis (VL), the Brazilian Visceral Leishmaniasis Control and Surveillance Program promotes the diagnosis and treatment of cases, vector control, euthanasia of seropositive dogs, and health education. Nevertheless, the effectiveness of these measures is questionable as they lead to little reduction in the transmission of the disease. Thus, the effectiveness of strategies such as insecticide-impregnated collars, spot-on insecticides, and immunization of dogs should be assessed. Herein, we evaluated the effectiveness of deltamethrin-impregnated collars on reducing the incidence of *Leishmania infantum* infection in dogs living in an endemic area of VL. An intervention study was conducted and a total 5,850 dogs were analyzed in baseline. Of these 3,742 seronegative dogs were divided into two groups: collared and uncollared (control). Dogs were followed for 12 months and three interventions were performed. The Cox regression model was used to evaluate the effectiveness of the collar. All analyzes were performed by Intention-to-treat and per-protocol. By intention-to-treat, the incidence rates of *L*. *infantum* infection were 7.5 and 7.9 in the collar group, and 6.5 and 13.2 per 1,000 dogs-months in the control group after 6 and 12 months, respectively. In the per-protocol analysis, the incidence rates in the control group were similar to those observed in the intention-to-treat analysis. In the collar group, the incidence rate was 5.1/1,000 dogs-months after 6 and 12 months. The effectiveness by intention-to-treat after adjustment by the multivariate Cox model was 48%. In the analysis per-protocol, the effectiveness increased to 63%. Although collar use was effective when it was evaluated by intention-to-treat, higher effectiveness was found in the per-protocol analysis after one year of follow-up. The data emphasize the importance of the uninterrupted use of deltamethrin-impregnated collars to increase protection against canine VL.

## Introduction

Visceral leishmaniasis (VL) is a neglected disease and, despite its worldwide distribution, 90% of global VL cases are concentrated in seven countries: Brazil, Ethiopia, India, Kenya, Somalia, South Sudan, and Sudan [1]. In the Americas, VL is present in 12 countries, with 96% of the cases being reported in Brazil [2]. In Brazil, VL is caused by the protozoan parasite *Leishmania infantum* and its most epidemiologically important vector is the sand fly *Lutzomyia longipalpis*. The transmission cycle in Brazil is zoonotic, with domestic dogs being the main urban reservoirs of the parasite [3].

The estimated annual incidence of VL in Brazil is 4.2 to 6.3 cases per 1,000 people per year, and case-fatality rate is around 7% [4]. Aiming at reducing morbidity and mortality rates, the Brazilian Ministry of Health has implemented the Visceral Leishmaniasis Control and Surveillance Program (VLCSP) to promote health education, early diagnosis and treatment of human VL cases, vector control with insecticides, and serological screening and euthanasia of infected dogs [5]. Recently, the treatment of canine visceral leishmaniasis (CVL) with miltefosine has been authorized by the Brazilian Ministries of Health and of Agriculture, Livestock and Supply. However, the decision to use this treatment is in the hands of dog owners’ as it has not been adopted as a large-scale preventive strategy in public health [6].

Despite the efforts of the VLCSP, the effectiveness of their actions remains questionable with regards to reduction of the transmission of the disease [7]. Studies evaluating the effectiveness of control measures such as dog-culling and spraying of residual insecticide, alone or in combination, suggested that these measures have been unsuccessful in controlling the disease [8–9]. Therefore, complementary measures such as the vaccination of dogs against leishmaniasis and/or the use of repellents in dogs are desirable. Leish-Tec is the only commercial canine vaccine against CVL licensed in Brazil [10], and its effectiveness in reducing the incidence of VL in humans is yet to be demonstrated. Thus, the vaccine is currently restricted to individual use and is not part of the strategies adopted by the VLCSP [11].

Previous experimental studies have shown that the use of topical insecticide and deltamethrin-impregnated collars can protect the dogs from phlebotomine bites [12–15], thus reducing canine *L*. *infantum* infection and indirectly preventing the human disease [16]. Nevertheless, these and similar studies in kenneled dogs [17–19] have been restricted to a small number of animals and/or specific conditions and do not reflect the effectiveness of the large-scale implementation of the collar in combination with other control actions, such as those performed by the VLCSP. Reithinger et al. [20] did not observe effectiveness of the collar in reducing the CVL incidence rates but, mathematical modelling of their results suggested that the use of dog collars may be a better alternative to euthanasia [20]. Hence, despite the potential of insecticide-impregnated collars to help control CVL, the effectiveness of its implementation in the context of VL control programs remains unknown.

Herein we evaluated the effectiveness of the large-scale implementation of deltamethrin-impregnated dog collars on reducing the incidence rate of *L*. *infantum* canine infection in an endemic area of VL. We performed our study within the context of the VLCSP in Brazil and demonstrated the effectiveness of the collars using two methods of analysis: by intention-to-treat and per-protocol.

## Materials and methods

### Ethical considerations

This study was approved by the Committee of Ethics in Animal Experimentation of the Universidade Federal de Ouro Preto (protocol no. 2014/18). All procedures followed guidelines set forth by the Brazilian Animal Experimental College (federal law number 11794). The owners of all the dogs taking part in this project were informed of the research objectives and signed a consent form prior to all procedures.

### Study area

**Fig 1:**
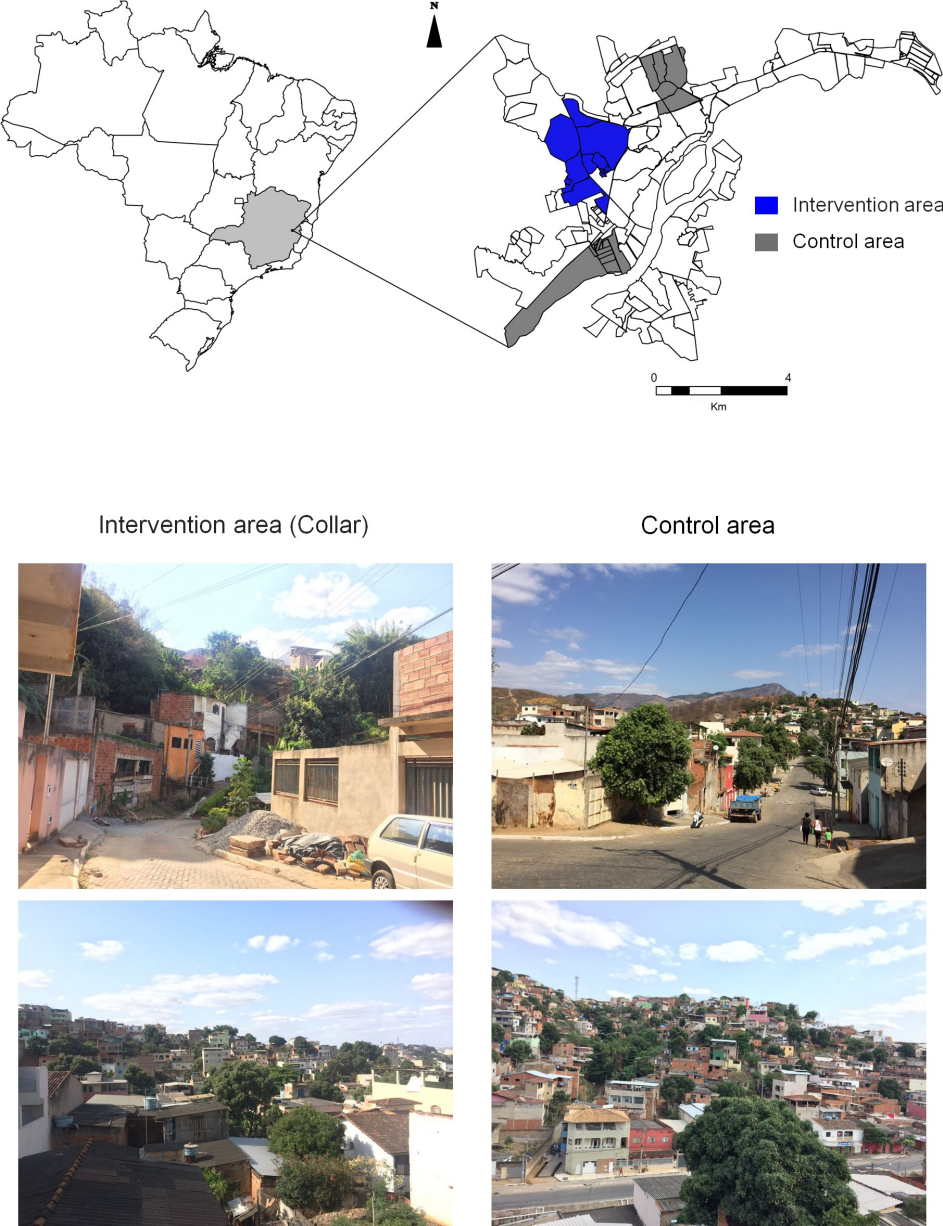
Map of the neighborhoods of the urban area of Govenador Valadares, Minas Gerais state, Brazil. In grey and blue are those neighborhoods selected for the Control and Intervention areas respectively.

The study was conducted in the municipality of Governador Valadares (18°51′04″ S, 41°56′58″ W), located in the eastern region of the state of Minas Gerais, within the Vale do Rio Doce region, in the southeast of Brazil (Fig 1). According to the Brazilian Institute of Geography and Statistics, the human population of this area is of 279,665 [21]. The city is endemic for VL and classified as an area of intense transmission by the Brazilian Ministry of Health [5]. Additionally, the area shows a high prevalence of CVL and a high density of the vector *L*. *longipalpis* [22]. Anti-rabies campaigns conducted by the Centre for Zoonosis Control of Governador Valadares have estimated the canine population to be approximately 23,000.

### Trial design

The intervention study was carried out from August 2014 to December 2015 in two regions of Governador Valadares (control and intervention) (Fig 1). The study design comprised one survey and three interventions, with a follow-up of 12 months. The intervention area and the control area were selected based on similar rates of canine infection prevalence by *L*. *infantum*, previously determined by the Municipal Health Department. Dogs present in each area were selected to be part of the group who received the collar (intervention) and the group that did not receive the collar (control). Therefore, the study areas were selected for convenience. The areas evaluated have high prevalence of CVL, human cases and high density of the vector *L*. *longipalpis* [22]. In addition, the selected areas were classified as of intense transmission (average of human cases in the last 5 years ≥ 4.4 cases) according to the Brazilian Ministry of Health [5]. The dogs of collar and control groups were selected from different areas to avoid that the control animals could be contaminated by the collars’ repellent.

The study procedures consisted of an interview with the owners, dog blood collection for serological tests, and collar change of dogs of the intervention area performed every six months. With the close collaboration of the Municipal Health Department, samples and data were collected during the VLCSP routine canine survey census conducted by the health agents.

The sample size was estimated using a 95% confidence interval (CI), 80% power, an exposed and unexposed ratio equal to one, and a 50% expected effectiveness of the collar. A total of 1,900 dogs should be included in each group. Thus, 3,835 households were assessed, and all dogs within the selected houses were sampled.

An initial survey (baseline) was conducted to select the seronegative dogs. Animals were screened through the serological test Dual Path Platform (DPP) and those seropositive were evaluated with the confirmatory test Enzyme Linked Immunosorbent Assay (ELISA). The Brazilian Ministry of Health recommends performing ELISA only for dogs seropositive by DPP and euthanasia of dogs positive by both DPP and ELISA. In the present study, however, dogs that were positive by DPP and negative by ELISA were also excluded from the study (i.e., only dogs negative by DPP were included).

The interventions evaluated the use of deltamethrin-impregnated collars, which consisted of plastic bands of 65 cm (25 g) containing 1 g of deltamethrin (Scalibor MSD Saúde Animal). In the first intervention (I), dog owners were interviewed by health agents using a structured questionnaire, and collars were placed on the dogs after the result of the serological test. After six months, in the second intervention (II), the collars were replaced, as determined by the manufacturer and another interview with the dog owners was performed before dog blood collection and serological tests were performed. Animals seropositive for both serological tests (DPP and ELISA) were euthanized as recommended by the VLCSP [5]. During intervention III, the procedures described in the intervention II were repeated, except for the collar replacement. During the interventions II and III, the dogs were evaluated regarding the use of the collar (i. e., if the dogs were still wearing the collars when health agents returned), the presence of any adverse reactions (as reported by the owners), and the history of vaccination against CVL. The information was registered on the forms, which were later digitized and analyzed. The same procedures (except for the replacement of collars) were performed for the control group.

The outcome evaluated in both control and collar groups were canine infection by *L*. *infantum* detected by both DPP and ELISA.

### Serological tests

For the serological tests, approximately 5 mL of venous blood were collected from the radial vein of large dogs and from the jugular vein of medium and small-sized dogs using 25x7 mm needles and sterile syringes. The material was sent to the Serology Laboratory of the Centre for Zoonosis Control of Governador Valadares for analysis.

Each sample was tested using the diagnostic protocol established by the Brazilian Ministry of Health[23]. The serology DPP CVL rapid test (Bio-Manguinhos/Fiocruz, Rio de Janeiro, Brazil) was used as a screening test, and ELISA (Canine Leishmaniasis EIE Kit/Bio-Manguinhos/Fiocruz, Rio de Janeiro, Brazil) was used as a confirmatory test. The diagnostic tests were carried out using sera samples, following the manufacturer’s instructions.

### Data collection

In the two studied areas (collared and uncollared), the health agents interviewed the dog owners using a previously tested structured questionnaire. Information was collected regarding the presence of previous cases of CVL; the spraying of insecticide by the VLCSP control program; the socioeconomic conditions and the educational level of dog owners; and the characteristics of the domicile and any adjacent backyards. In addition, the following information was collected about the dogs: age, gender, size, hair type, vaccination and veterinary checkups, behavior (the place where the dog slept and spend most of its time), and clinical data. The information obtained in the questionnaire was used to adjust the estimation of the effectiveness of the collar.

### Statistical analysis

The collected information was double entered into the EpiData software version 3.2 (EpiData Association, Odense, Denmark). The double entries were compared, and any identified divergencies were rectified. The database consistency was tested, and summary measures of the analysis were made. Statistical analyzes were performed using STATA version 15.0 (Stata Corp., College Station, TX, USA). We verified the comparability between the collar and control groups using the chi-square test.

Although the current study is not a randomized clinical trial, all analyzes were performed by intention-to-treat and per-protocol, as recommended by the CONSORT guidelines [24]. Intention-to-treat analyses include all individuals taking part in the study regardless of noncompliance, protocol deviations, withdrawal, and any other incidents that may happen after randomization. On the other hand, per-protocol analyses include the population that completed the study without any major protocol violations [25]. Thus, in our intention-to-treat analysis, we compared all dogs allocated to the groups (collared and control), regardless of whether they were using the collar at the time of interventions II and III. In the per-protocol analysis, only animals that had the collar throughout the duration of the study were compared with controls.

The person-time incidence rate was estimated as the ratio between the number of new cases of canine *L*. *infantum* infection over person-time in the follow-up (12 months), per group evaluated (collar and control). The denominator was the time of follow-up of dogs evaluated throughout the year plus half of the follow-up time of the dogs which were lost during the follow-up.

The Cox regression model was carried out using the “stcox” function in STATA to evaluate the effectiveness of the collar adjusted by the variables obtained in the questionnaire. Univariate analysis was performed and variables that showed statistical association (p < 0.25) were selected to compose the multivariate model. The building of the final models started with the full models, containing all variables (p < 0.25), followed by successive discarding (backward selection) of the non-significant variables. The final multivariate regression models to evaluate effectiveness was adjusted for variables that remained statistically significant (p<0.05), thereby taking into account the effects of these variables on the incidence of seroconversion for CVL. Variables with more than two categories were transformed into *dummy* variables. The strength of association was determined by hazard ratios (HRs) at a 95% CI. In addition, the multivariate model was adjusted by variables of the questionnaire to control for potential confounder factors.

## Results

### Phases of the study

**Fig 2:**
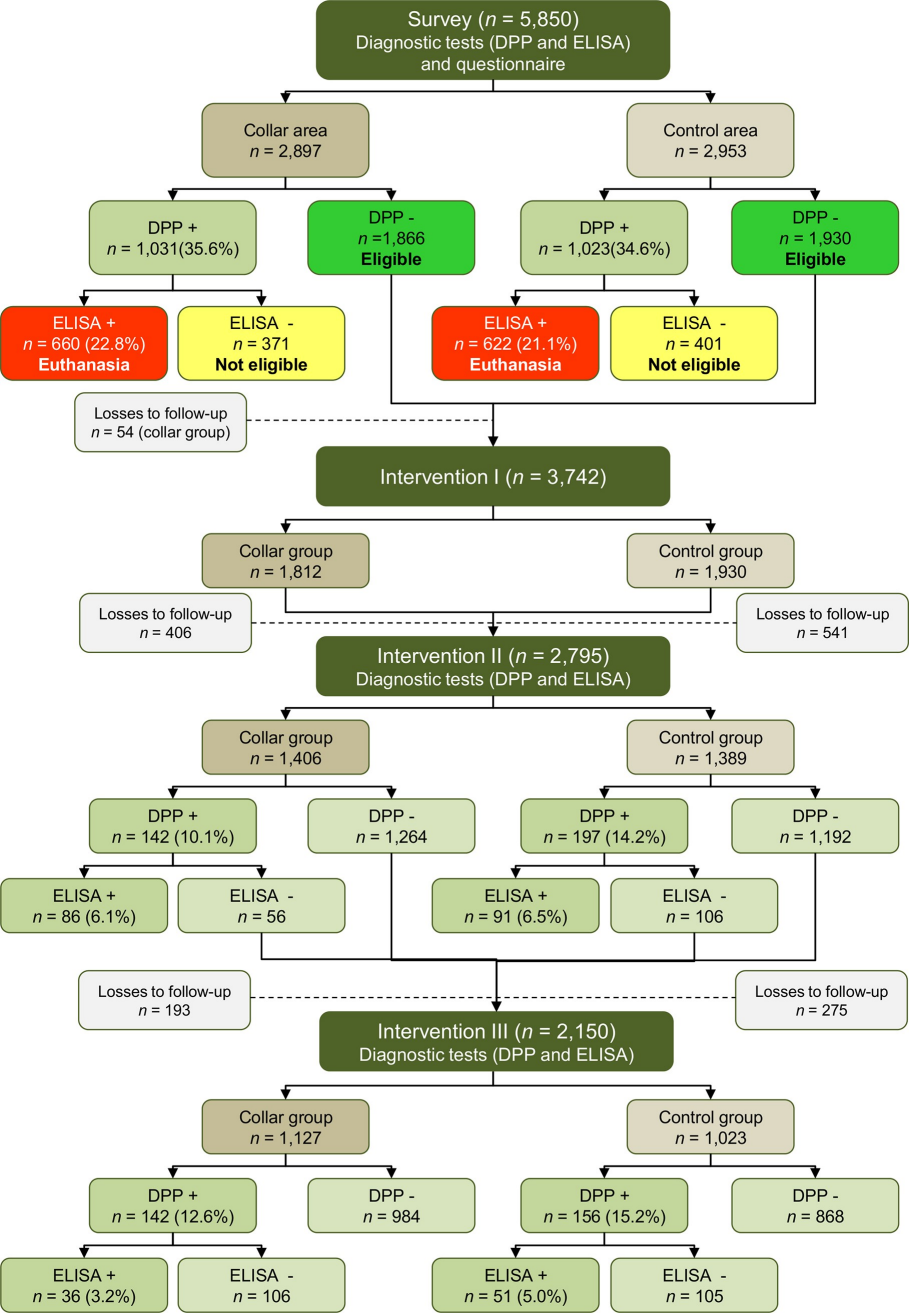
Schematic representation of the phases of the study and the serological test results.

A schematic representation of the phases of the study and the serological tests results is presented in Fig 2.

#### Survey

The survey was conducted from August 2014 to February 2015. During this period, samples of 5,850 dogs were collected: 2,897 in the area where dogs would receive the collars (collared area) and 2,953 in the control (uncollared) area. Among these animals, 1,031 (35.6%) in the collar area and 1,023 (34.6%) in the control area were DPP positive. The prevalence of infection in each area was determined following the confirmatory test (ELISA). The prevalence of *L*. *infantum* canine infection in the collar area was 22.8% (95% CI 21.3–24.3). A similar prevalence was observed in the control area, 21.1% (95% CI 19.6–22.6). Animals that presented discordant serological results were excluded from the study. Thus, 1,866 dogs in the collar area and 1,930 dogs in the control area were negative by DPP and eligible for the intervention study.

#### Intervention I

The interventions started in September 2014, after the results of the serological tests. From the 3,796 animals eligible for the study (1,866 in the collared group and 1,930 in control group), 54 (collared group) were not found at the time of the intervention I. Therefore, 1,812 animals in the collared group and 1,930 in the control group took part in this first intervention.

#### Intervention II

Intervention II was carried out between March and July 2015. In this intervention, 406 (22.4%) dogs were lost to follow-up in the collared group and 541 (28.0%) in the control group. Therefore, 1,406 dogs were evaluated in the collared group and 1,389 in the control group. The incidence of infection by *L infantum* was 6.1% in the collared group and 6.5% in the control group.

#### Intervention III

The last intervention was carried out between September and December 2015. In this intervention, 193 (14.6%) dogs were lost to follow-up in the collared group and 275 (21.2%) in the control group. Therefore, 1,127 dogs were evaluated in the collar group and 1,023 in the control group. The incidence of infection was 3.2% in the collared group and 5.0% in the control group (Fig 1).

### Characteristics of the dogs and housing conditions

In both collared and control groups, most of the dogs were small-sized females with short fur, which were not taken to the veterinarian for check-up, spent most of the time and slept in the backyard, did not have access to the street, and did not use shampoo against flea and tick (Table 1). A significant difference was observed between the groups regarding size, veterinary check-ups, access to the street, and the use of shampoo against flea and tick. These differences were approximately 2–8% for all variables, except the use of shampoo against flea and tick (Table 1).

**Table 1:** Characteristics of the dogs in the collared and control groups and evaluation of the comparability between groups. Governador Valadares, Minas Gerais, Brazil, 2014–2015.

Variable	Collared group n (%)		Control group n (%)		*P*
**Collar**					
No	–		1,930		
Yes	1,812		–		
**Sex**					
Male	843 (46.5)		857 (44.4)		
Female	969 (53.5)		1073 (55.6)		0.193
Size*					
Small	1,009 (55.7)		1,113 (57.7)		
Medium	663 (36.6)		610 (31.6)		
Big	140 (7.7)		207 (10.7)		0.001
**Fur length**					
Long	648 (35.8)		721 (37.4)		
Short	1,164 (64.2)		1,209 (62.6)		0.311
Veterinary check-ups*					
Yes	305 (16.8)		441 (22.9)		
No	1,506 (83.2)		1,488 (77.1)		0.001
**Place where dogs lived and rested**					
Inside the house	302 (16.7)		323 (16.7)		
In the backyard	929 (51.3)		990 (51.3)		
In the balcony	581 (32.0)		617 (32.0)		0.997
**Sleeping place**					
Inside the house	291 (16.1)		335 (17.4)		
In the backyard	948 (52.3)		983 (50.9)		
In the balcony	573 (31.6)		612 (31.7)		0.542
Had access to the street*					
No	1,273 (70.3)		1,508 (78.1)		
Yes	538 (29.7)		422 (21.9)		0.001
Shampoo to flea and tick*					
No	1,240 (68.4)		1,045 (54.2)		
Yes	572 (31.6)		882 (45.8)		0.001

* Variables with significant difference between collared and control groups.

We observed that 198 (5.3%), 163 (5.8%), and 83 (3.8%) dogs presented symptoms compatible with CVL in interventions I, II, and III respectively. The most frequently observed symptoms were: onychogryphosis and lesion on the skin, ears, muzzle, and joints (S1 Table). Considering that the dogs were not palpated by a veterinary, the presence of lymphadenopathy, hepato and splenomegaly in the animals was not quantified.

A total of 2,494 households were evaluated: 1,218 in the collared area and 1,276 in the control area. Both areas presented a similar distribution of dogs per residence, with a mean of 1.5 and standard deviation of 0.9 dogs per household (1–10 dogs/house) and median of 1 dog per household with interquartile range of 1–2. Most of the households were owned or financed by the residents, had no history of CVL cases, and had cemented backyards. Few backyards had plant beds, banana trees, garbage, dry leaves, trees, or manure. Regarding dog owners’ schooling, the collared area had mainly owners who had completed primary school, while in the control area most owners had finished secondary school/university. Both areas were predominantly composed of socioeconomic class C1 (Table 2). Differences were observed between the collared and control areas for all the housing and socioeconomic conditions, except for the “presence of backyard” and the “presence of plant beds in the backyard”.

**Table 2:** Characteristics of the owners, domicile and peridomicile of the dogs in the collared and control groups evaluation of the comparability between groups. Governador Valadares, Minas Gerais, Brazil, 2014–2015. (Information obtained from 2,494 households).

Variable	Collared group n (%)	Control group n (%)	*P*
**Schooling**			
Illiterate	422 (34.7)	412 (32.3)	
Primary school	478 (39.3)	371 (29.1)	
Secondary School/University	317 (26.0)	492 (38.6)	0.001
**Home**			
Own or financed	1,083 (89.0)	1,025 (80.3)	
Rented	134 (11.0)	251 (19.7)	0.001
Socioeconomic class*			
A2/ B1	36 (3.0)	74 (5.8)	
B2	186 (15.3)	314 (24.6)	
C1	508 (41.8)	478 (37.4)	
C2/D/E	485 (38.9)	410 (32.2)	0.001
**Previous case of CVL in the household**			
No	784 (64.4)	971 (76.1)	
Yes	434 (35.6)	305 (23.9)	0.001
**Backyard**			
No	381 (31.3)	442 (34.6)	
Yes	837 (68.7)	834 (65.4)	0.075
**Backyard features**			
Cement	348 (41.5)	382 (45.8)	
Ground	208 (24.8)	155 (18.5)	
Cement and ground	282 (33.7)	298 (35.7)	0.008
**Plant bed in the backyard**			
No	538 (44.2)	566 (44.4)	
Not applicable	381 (31.3)	442 (34.6)	
Yes	299 (24.5)	268 (21.0)	0.061
**Banana tree in the backyard**			
No	707 (58.0)	734 (57.5)	
Not applicable	381 (31.3)	442 (34.6)	
Yes	130 (10.7)	100 (7.9)	0.022
**Garbage in the backyard**			
No	535 (43.9)	700 (54.9)	
Not applicable	381 (31.3)	442 (34.6)	
Yes	302 (24.8)	134 (10.5)	0.001
**Dry leaves in the backyard**			
No	466 (38.2)	670 (52.5)	
Not applicable	381 (31.3)	442 (34.6)	
Yes	371 (30.5)	164 (12.9)	0.001
**Trees in the backyard**			
No	313 (25.7)	358 (28.1)	
Not applicable	381 (31.3)	442 (34.6)	
Yes	524 (43.0)	476 (37.3)	0.014
**Manure in the backyard**			
No	790 (64.8)	747 (58.5)	
Not applicable	381 (31.3)	442 (34.6)	
Yes	47 (3.9)	87 (6.8)	0.001

* A2/ B1 = class that receives more than 10 minimum wages.

B2 = class that receives between 6 and 10 minimum wages.

C1 = class that receives between 4 and 6 minimum wages.

C2/D/E = class that receives less than 4 minimum wages.

Brazilian monthly minimum wage = R$788.00 / ~ U$329.

### Losses to follow-up

In total, 947 (25.3%) and 468 (16.7%) dogs were lost to follow-up in interventions II and III, respectively. The reasons for losses to follow-up were change of address, dog absence due to donation, death or escape, closed household, or the owners’ refusal to continue in the study. Households were considered lost to follow-up after three unsuccessful attempts to contact the owners. The size of the animals varied among the dogs that were lost to follow-up in the two groups (collar and control). Loss to follow-up was higher among small-sized animals in both groups: 52.8% in the collared group and 57.2% in the control group. In addition, there was variation in the loss of follow-up between the groups in the variables access to the street and use of shampoo for flea and tick (S2 Table). When comparing the dogs that were lost in follow-up with maintained in the study, differences were observed in size, veterinary check-ups, the place where the animal slept or spent most of its time and access to the street by the dog (S3 Table).

### Incidence of dog-time

Among the 2,795 dogs evaluated by intention-to-treat in intervention II, 86 were positive by DPP and ELISA in the collared group (n = 1,406) and 91 in the control group (n = 1,389). The overall incidence rates were 7.5 (95% CI 5.1–6.5) and 7.9 (95% CI 5.1–6.5) per 1,000 dog-months during six months in the collared and control groups, respectively. In intervention III (2,150 dogs), the incidence rates were 6.5 (95% CI 4.7–9.0) and 13.2 (95% CI 10.0–17.3) per 1,000 dog-months during 12 months in the collared and control groups, respectively (Table 3).

**Table 3:** Incidence dog/time of infection by *Leishmania infantum* by intention-to-treat and by per-protocol analysis, Governador Valadares, Minas Gerais, Brazil, 2014–2015.

Analysis and Intervention		Group			
		Collared		Control	
		Failure events^c^	Incidence rate^d^ (95% CI)	Failure events^c^	Incidence rate^d^ (95% CI)
Intention-to-treat	II^a^	86	7.5 (6.1–9.4)	91	7.9 (6.4–9.7)
	III^b^	36	6.5 (4.7–9.0)	51	13.2 (10.0–17.3)
Per-protocol	II^a^	35	5.1 (3.6–7.1)	91	7.9 (6.4–9.7)
	III^b^	16	5.1 (3.1–8.3)	51	13.2 (10.0–17.3)

^a^ Intervention II, performed after approximately six months of the first intervention.

^b^ Intervention III, performed after approximately 12 months of the first intervention.

^c^ Failure events = seroconversion (DPP and ELISA positive).

^d^ Incidence rate/1000 dogs-months.

Among the 1,406 dogs in the collared group, 920 were using the collar in intervention II (losses of 34.5%). Per-protocol analysis of these dogs revealed an incidence of 5.1 (95% CI 3.6–7.1) per 1,000 dog-months during six months in the collared group. In intervention III, among the 1,127 dogs that finished the study in the collared group, 828 were using the collar (losses of 26.5%). However, in the per-protocol analysis, only dogs who kept the collar throughout the study (n = 632) were considered. The incidence among these dogs in intervention III was 5.1 (95% CI 3.1–8.3) per 1,000 dog-months during 12 months. For both interventions II and III, the per-protocol incidence in the control group was the same as that observed in the intention-to-treat analysis, as the same animals were considered in both analyses (Table 3).

### Effectiveness and adverse effects of the deltamethrin-impregnated collars

To estimate the effectiveness by intention-to-treat and per-protocol, we first performed a univariate analysis (S4 and S5 Tables). The multivariate Cox final model in the intention-to-treat analysis showed an HR of 0.52 (95% CI 0.40–0.68) when adjusted for place where dogs lived and rested; the dogs’ accessibility to the street; and the use of shampoo against flea and ticks. In this analysis, the effectiveness of the collar was 48%. On the other hand, the per-protocol analysis revealed an HR of 0.37 (95% CI 0.26–0.52) when adjusted for the presence of dry leaves in the backyard, place where dogs sleeps, and the use of shampoo against flea and ticks. In this analysis, the effectiveness of the collar raised to 63% (Table 4).

**Table 4:** Effectiveness of deltamethrin-impregnated collars in controlling canine infection by *L*. *infantum* according to the final Cox regression model. Governador Valadares, Minas Gerais, Brazil, 2014–2015.

Collar	Intention-to-treat			Per-protocol		
	HR (95%CI)^a^	HR (95% CI)^b^	Effectiveness^d^	HR (95%CI)^a^	HR (95%CI)^c^	Effectiveness^d^
No *versus* Yes	0.63 (0.49–0.81)	0.52 (0.40–0.68)	48%	0.44 (0.32–0.61)	0.37 (0.26–0.52)	63%

^a^HR crude

^b^HR adjusted for place where dogs lived and rested; accessibility to the street; and the use of shampoo against flea and tick

^c^HR adjusted for dry leaves in the backyard; place where the dog sleeps; and the use of shampoo against flea and tick

^d^Effectiveness = (1-HR_adjusted_)x100

Adverse effects to the collar were registered for 62 dogs (4.4%) in intervention II (reflecting the adverse effects due to intervention I), and for 43 dogs (3.8%) in intervention III (indicating the adverse effects due to intervention II). The adverse effect most frequently reported by the dog owners was local allergic reaction.

## Discussion

The intention-to-treat analysis showed a 48% effectiveness of deltamethrin-impregnated collars within an area of high incidence of *L*. *infantum* infection. The per-protocol analysis revealed that the collars’ effectiveness raised to 63%. According to Giorgi Rossi [26], per-protocol analysis shows the effectiveness of the collars under ideal or experimental conditions; i.e., the protection that collars would provide if the population fully adhered to the procedures of adequately collaring dogs. However, in the context of public health, the results from the intention-to-treat analysis are more representative of the real impact of the use of the collars, as the most important obstacle to achieving effectiveness is low adherence [27]. Our data emphasize the importance of the uninterrupted use of the collars for large-scale protection against CVL. Regarding safety, the collar was considered safe, presenting a low rate of mild adverse reactions (< 4.5%).

A recent study conducted in Brazil showed that the main problems faced by public health managers to implement VL control strategies in large urban centers are: (i) the discontinuity of activities due to lack of financial, material, or human resources; (ii) refusal of the population to allow blood collection in dogs and chemical control; and (iii) owners’ resistance to dogs’ euthanasia [28]. Euthanasia of seropositive dogs is justified by the high cutaneous parasitism of the animals, regardless of the clinical form, which contributes to the maintenance and expansion of the disease transmission cycle [29–31]. However, the effectiveness of this strategy in reducing the incidence of the disease is questionable [32–33]. These controversies led to its rejection by the community [28] and delayed action and consequently reduced the practical effectiveness of dog euthanasia [34]. Therefore, the search for strategies with better acceptability is necessary. A viable alternative for controlling CVL is the prevention of sandfly bites using pyrethroid insecticide formulations [15, 17, 35–36]. The results from our large-scale intervention reinforce the viability of using insecticide-impregnated collars as an alternative strategy in VL control programs such as the VLCSP.

Previous studies have shown that in controlled environments (kennels) and/or in small populations located in endemic areas, deltamethrin-impregnated collars are useful in controlling the infection by *L*. *infantum*, with protection ranging from 50% to 84% [17, 19, 35]. One study conducted with 120 dogs (60 collared and 60 uncollared) in an open-air kennel in an endemic area of Italy showed a 50.8% protection after two years of monitoring [17]. Ferroglio et al. [35] observed an 84% reduction in canine infection rates by *L*. *infantum* using collars impregnated with deltamethrin and a spot-on solution of permethrin in a kennel of another endemic area in Italy. Two types of insecticide-impregnated collars (Seresto and Scalibor) used in 224 dogs housed in private shelters showed an efficacy of 88.3% in the group using Seresto collars and 61.8% in the group using the Scalibor, after one year of follow-up [19]. A field study in Iran noted the effectiveness of collar in reducing cases of VL in children and dogs [16]. The results presented herein corroborate and expand upon these data since we analyzed a larger sample in an important endemic area in Brazil that lacked this information. To the best of our knowledge, no study of this magnitude evaluating the use of the collar concomitantly with the actions of a VL control program has been published.

Herein we evaluated a large number of dogs and assessed the effectiveness of a large-scale VL control intervention under real-life conditions. Our results reflect the effectiveness of the deltamethrin-impregnated collars combined with the other strategies currently adopted by the VLCSP. The improved effectiveness of these collars that we observed after one year of follow-up (in comparison with the follow-up for six months) was expected, as it possible that false-negative dogs may have been included at the beginning of the study. In the early stages of the disease, there is an immunological window period in which the antibody titer secreted by B lymphocytes is very low, which leads to lower sensitivity and higher false-negative rates in the serological tests. Considering that seroconversion may occur between 1 and 6 months after experimental infections [37], and in approximately 10.5 months (range, 4–22 months) following natural infection [38], it is possible that infected dogs with false-negative results in the first DPP test seroconverted between our interventions. Given the longer immunological window observed after the natural infections, it is possible that the observed effectiveness would be even higher after longer periods of follow-up. Consistent with this findings, a field evaluation with 350 dogs showed a 50% efficiency of deltamethrin-impregnated collars in the first year and 86% in the second year [36], thus demonstrating that effectiveness increases with the duration of the intervention.

Although the intention-to-treat analysis is the method recommended to avoid bias, our per-protocol analysis revealed the impact of the continuous use of the collar on its effectiveness, with increased effectiveness being observed among the dogs that kept the collar throughout the follow-up. These data highlight the importance of raising awareness of dog owners for responsible pet ownership and persistent placement of collars in their dogs to increase the effectiveness of this VL control strategy. Moreover, to maintain effectiveness, VL control programs should adopt strategies for replacing lost collars.

The households evaluated in this study were sampled from a census survey involving areas representative of the entire municipality of Governador Valadares, thus increasing the external validity of the study. One limitation of the study was the attrition rate due to losses of follow-up. A comparison of several features indicated that the dogs lost to follow-up differed from those that remained in the study regarding size, veterinary check-ups, the place where animals slept or spent most of their time, and access to the street by the dog. However, the effect of these differences may be minimal, as variations in each category of these variables were small (< 5%). In addition, these differences were also observed among the characteristics of the dogs of the two evaluated areas. Also, we believe that the adjustment of the model minimized these distortions. Despite some differences observed between the areas, the prevalence rates of canine infection were similar, which increases the comparability between the regions. The exclusion of a considerable number of animals in our per-protocol analysis may have affected our results by inserting group comparison bias and reducing sample size and the power of the study [39]. In addition, per-protocol analysis may have excluded dogs from less careful owners (who allowed the animals to lose their collars), thus leading to bias. On the other hand, these results provided an estimate of the true efficacy of the intervention among dogs that kept the collar throughout the study and reinforce the importance of the collar’s persistent and correct use to grant higher effectiveness.

One possible issue that may arise during the implementation of deltamethrin-impregnated collars as an auxiliary strategy to control VL, is the high number of stray dogs in Brazil. Thus, the use of collars in domiciled dogs only may be insufficient to contain the disease. Therefore, castration can be a complementary action to reduce the number of stray dogs and contribute to the control of VL, since the number of infected reservoirs is correlated with the increased risk of human disease [40]. Noteworthy, the effectiveness of the collars is most pronounced in urban areas, where the dog is the main reservoir of the disease, and where almost 80% of the human VL cases in Brazil are registered [41]. The implementation of deltamethrin-impregnated collars by VL control programs and concomitant complementary strategies should be adopted to ensure success.

## Conclusions

The data presented herein indicates that deltamethrin-impregnated collars are effective for reducing canine VL infection after one year of follow-up and suggest a large-scale implementation of these collars as a VL control strategy that could produce great results in the medium and long terms.

## Supporting information

S1 Checklist TREND checklist. (PDF)

S1 Database Database with variables used in the analysis of this study. (XLSX)

S1 Appendix Questionnaire. (DOCX)

S1 Table Symptoms compatible with canine visceral leishmaniasis. (DOCX)

S2 Table Characteristics of the dogs in the collared and control groups that were lost during follow-up. (DOCX)

S3 Table Characteristics of dogs in follow-up and of those lost during follow-up. (DOCX)

S4 Table Univariate analysis of the characteristics of the dogs. (DOCX)

S5 Table Univariate analysis of the characteristics of owners, domicile and peridomicile of the dogs. (DOCX)
