# Supplementary material for: Effectiveness of deltamethrin-impregnated dog collars on the incidence of canine infection by Leishmania infantum: A large scale intervention study in an endemic area in Brazil
Source: PLoS One. 2018 Dec 10;13(12):e0208613. doi: 10.1371/journal.pone.0208613 (PMC6287856; doi:10.1371/journal.pone.0208613)
Supplement: S1 Appendix — (DOCX) [file pone.0208613.s003.docx]

| Questionnaire | Codification |
| --- | --- |
| Date: ____/______/________ |  |
| Addrees:_____________________________________________________________________________________________________________________  Owner name: __________________________________________________  Telephone: _____________ Cell phone: ____________________________ | Bairro  Rua  propri |
|  |  |
| Previous case of CVL in the household  0 ( ) No 1 ( ) Yes 9 ( ) Did not answer | CAOLVC |
| Has this house been sprayed with insecticide to control leishmaniasis?  0 ( ) No 1( ) Yes 7 ( ) Do not know 9( ) Did not answer | BORRIF |
| Is there a backyard in the residence?  0 ( ) No 1 ( ) Yes 9 ( ) Did not answer | QUINTAL |
| What are the features of the backyard?  0 ( ) Cement 1( ) Ground 2( ) Cement and ground 8 ( ) No applicable | CQUINT |
| What is in the backyard of this residence?  0 ( ) No 1 ( ) Yes 8 ( ) No applicable  **a) HAQCP** ( ) Plant bed  **b) HAQBA** ( ) Banana tree  **c) HAQLX** ( ) Garbage  **d) HAQMO** ( ) Dry leaves  **e) HAQAR** ( ) Trees  **f) HAQES** ( ) Manure | a. HAQCP  b. HAQBA  c. HAQLX  d. HAQMO  e. HAQAR  f. HAQES |
| How many color televisions are in the residence?  0 ( ) 1 ( ) 2 ( ) 3 ( ) 4 ou + ( ) | TV |
| How many radios are there in the residence?  0 ( ) 1 ( ) 2 ( ) 3 ( ) 4 ou + ( ) | RADIO |
| Quantos banheiros há na residência?  0 ( ) 1 ( ) 2 ( ) 3 ( ) 4 ou + ( ) | BANHEIRO |
| How many bathrooms are there in the residence?  0 ( ) 1 ( ) 2 ( ) 3 ( ) 4 ou + ( ) | CARRO |
| How many employees are there in the residence?  0 ( ) 1 ( ) 2 ( ) 3 ( ) 4 ou + ( ) | EMPREG |
| How many washing machines are there in the residence?  0 ( ) 1 ( ) 2 ( ) 3 ( ) 4 ou + ( ) | MLAVAR |
| How many DVDs are there in the residence?  0 ( ) 1 ( ) 2 ( ) 3 ( ) 4 ou + ( ) | DVD |
| How many refrigerators are there in the residence?  0 ( ) 1 ( ) 2 ( ) 3 ( ) 4 ou + ( ) | GELAD |
| How many freezers (or part of the duplex refrigerator) is in the residence?  0 ( ) 1 ( ) 2 ( ) 3 ( ) 4 ou + ( ) | FREEZER |
| Schooling of the head of the family.  1 ( ) Illiterate  2 ( ) Primary school  3 ( ) Secondary School  4 ( ) University | ESCOLRE |
| Your home is:  1 ( ) Own 2 ( ) Rented 3 ( ) financed | CASA |

| Dog data |  |
| --- | --- |
| Owner name:_____________________________________________ | PROPRIFC |
| Group: 0 ( ) control 1 ( ) collar | GRUPO |
| When was the collar put on? ___/___/_____ | Coleira |
| DPP: 0 ( ) Negative 1 ( ) Positive | DPP1 |
| ELISA: 0 ( ) Negative 1 ( ) Positive 3 ( ) Unrealized | ELISA1 |
| Sample code: ______________ | COD________ |
| Name of dog:_____________________ Age:_______ Color:_____________ | Nome_________  Idade___________ |
| Sex: 0 ( ) Male 1 ( ) Female / Breed:________________ | SEXOCAO |
| Size: 0 ( ) Small 1 ( ) Medium 2 ( ) Big | PORTE |
| Fur length: 0 ( ) Long 1 ( ) Short | PELO |
| Do you take the dog to the vet?  0 ( ) No 1 ( ) Yes 7 ( ) Do not know 9( ) Did not answer | VETER |
| Place where dogs lived and rested  0 ( ) Inside the house 1 ( ) In the backyard 2 ( ) In the balcony | ONDFIC |
| Place where the dog sleeps  0 ( ) Inside the house 1 ( ) In the backyard 2 ( ) In the balcony | ONDDORM |
| Had access to the street?  0 ( ) No 1 ( ) Yes 7 ( ) Do not know 9( ) Did not answer | CFPGE |
| How long has the dog been living with the family in this residence?  1 ( ) months:___ 2( ) years:____ 7 ( ) Do not know 9( ) Did not answer | QTCMOR |
| Do you regularly use flea and tick shampoo on this dog?  0 ( ) No 1 ( ) Yes 7 ( ) Do not know 9( ) Did not answer | SHAMPOO |
| Does this dog show symptoms of visceral leishmaniasis?  0 ( ) No 1 ( ) Yes | APSINT |
| What symptoms do you notice? Check with “X” the symptoms you are seeing in the dog.  a) ( ) Alopecia h ( ) Keratoconjunctivitis  b) ( ) Onychogryphosis i ( ) Apathy  c) ( ) Opaque bristles j ( ) Weight Loss  d) ( ) Loss of appetite K ( ) Diarrhoea  e) ( ) Coriza L ( ) Vomiting  f) ( ) Splenomegaly  g) ( ) Lesion on the skin | a. QSINTPL  b. QSINTUN  c. QSINTPO d.QSINTCP  e QSINTCO  f. QSINTAB  g. QSINTMA  h. QSINTCJ  i. QSINTDM  j. QSINTEM  k. QSINTDI  l. QSINTVO |
| Was the dog wearing a collar on return?  0 ( ) No 1 ( ) Yes 8 ( ) No applicable | COLR1 |
| Has the animal had any adverse reaction to the collar?  0 ( ) No 1 ( ) Yes 8 ( ) No applicable  If so, which one?_________________________________ | REACR1 |
