# Supplementary material for: Effectiveness of deltamethrin-impregnated dog collars on the incidence of canine infection by Leishmania infantum: A large scale intervention study in an endemic area in Brazil
Source: PLoS One. 2018 Dec 10;13(12):e0208613. doi: 10.1371/journal.pone.0208613 (PMC6287856; doi:10.1371/journal.pone.0208613)
Supplement: S1 Table — (DOCX) [file pone.0208613.s004.docx]

**S1Table**: Symptoms compatible with canine visceral leishmaniasis.

|  | **Intervention I**  **n (%)** | **Intervention II**  **n (%)** | **Intervention III**  **n (%)** |
| --- | --- | --- | --- |
| Symptomatic dogs | 198/3,741 (5.3) | 163/1,812 (5.8) | 83/2,182 (3.8) |
| Symptoms |  |  |  |
| Onychogryphosis | 91 (46.6) | 61 (37.4) | 25 (30.1) |
| Lesion on the skin, ears, muzzle, and joints | 76 (38.4) | 57 (35.0) | 40 (48.2) |
| Weight Loss | 54 (27.3) | 41 (25.2) | 19 (22.9) |
| Alopecia | 43 (21.7) | 27 (16.6) | 13 (15.7) |
| Loss of appetite | 19 (9.6) | 15 (9.2) | 2 (2.4) |
| Apathy | 18 (9.1) | 20 (12.3) | 10 (12.0) |
