# Supplementary material for: Effectiveness of deltamethrin-impregnated dog collars on the incidence of canine infection by Leishmania infantum: A large scale intervention study in an endemic area in Brazil
Source: PLoS One. 2018 Dec 10;13(12):e0208613. doi: 10.1371/journal.pone.0208613 (PMC6287856; doi:10.1371/journal.pone.0208613)
Supplement: S2 Table — (DOCX) [file pone.0208613.s005.docx]

**S2 Table:** Characteristics of the dogs in the collared and control groups that were lost during follow-up.

| **Variable** | **Collared group**  **n (%)** | | **Control group**  **n (%)** | | ***P*** |
| --- | --- | --- | --- | --- | --- |
| **Collar** |  | |  | |  |
| No | − | | 816 | |  |
| Yes | 599 | | − | |  |
| **Sex** |  | |  | |  |
| Male | 291 (48.6) | | 371 (45.5) | |  |
| Female | 308 (51.4) | | 445 (54.5) | | 0.249 |
| **Size** |  | |  | |  |
| Small | 316 (52.8) | | 467 (57.2) | |  |
| Medium | 226 (37.7) | | 253 (31.0) | |  |
| Big | 57 (9.5) | | 96 (11.8) | | 0.024 |
| **Fur length** |  | |  | |  |
| Long | 222 (37.1) | | 292 (35.8) | |  |
| Short | 377 (62.9) | | 524 (64.2) | | 0.622 |
| **Veterinary check-ups** | |  | |  | |
| Yes | 91 (15.2) | | 154 (18.9) | |  |
| No | 508 (84.8) | | 662 (81.1) | | 0.071 |
| **Place where dogs lived and rested** |  | |  | |  |
| Inside the house | 80 (13.4) | | 125 (15.3) | |  |
| In the backyard | 333 (55.6) | | 409 (50.1) | |  |
| In the balcony | 186 (31.0) | | 282 (34.6) | | 0.124 |
| **Sleeping place** |  | |  | |  |
| Inside the house | 80 (13.4) | | 125 (15.3) | |  |
| In the backyard | 333 (55.6) | | 409 (50.1) | |  |
| In the balcony | 186 (31.0) | | 282 (34.6) | | 0.124 |
| **Had access to the street** | |  | |  | |
| No | 399 (66.6) | | 622 (76.2) | |  |
| Yes | 200 (33.4) | | 194 (23.7) | | 0.001 |
| **Shampoo to flea and tick** | |  | |  | |
| No | 415 (69.3) | | 460 (56.5) | |  |
| Yes | 184 (30.7) | | 354 (43.5) | | 0.001 |
