# Supplementary material for: Effectiveness of deltamethrin-impregnated dog collars on the incidence of canine infection by Leishmania infantum: A large scale intervention study in an endemic area in Brazil
Source: PLoS One. 2018 Dec 10;13(12):e0208613. doi: 10.1371/journal.pone.0208613 (PMC6287856; doi:10.1371/journal.pone.0208613)
Supplement: S3 Table — (DOCX) [file pone.0208613.s006.docx]

**S3 Table:** Characteristics of dogs in follow-up and of those lost during follow-up.

| **Variable** | **Dogs in follow-up**  **n (%)** | | **Losses to follow-up**  **n (%)** | | ***p*** |
| --- | --- | --- | --- | --- | --- |
| **Collar** |  | |  | |  |
| No | − | | 1,415 | |  |
| Yes | 2,150 | | − | |  |
| **Sex** |  | |  | |  |
| Male | 943 (43.9) | | 662 (46.8) | |  |
| Female | 1,207 (56.1) | | 753 (53.8) | | 0.086 |
| **Size** |  | |  | |  |
| Small | 1,259 (58.5) | | 783 (55.3) | |  |
| Medium | 715 (33.3) | | 479 (33.9) | |  |
| Big | 176 (8.2) | | 153 (10.8) | | 0.018 |
| **Fur length** |  | |  | |  |
| Long | 808 (37.6) | | 514 (36.3) | |  |
| Short | 1,342 (62.4) | | 901 (63.7) | | 0.447 |
| **Veterinary check-ups** | |  | |  | |
| Yes | 478 (22.2) | | 245 (17.3) | |  |
| No | 1,671 (77.8) | | 1,170 (82.7) | | 0.001 |
| **Place where dogs lived and rested** |  | |  | |  |
| Inside the house | 407 (18.9) | | 208 (14.7) | |  |
| In the backyard | 1,033 (48.1) | | 749 (52.9) | |  |
| In the balcony | 710 (33.0) | | 458 (32.4) | | 0.001 |
| **Sleeping place** |  | |  | |  |
| Inside the house | 409 (13.4) | | 205 (14.5) | |  |
| In the backyard | 1,054 (55.6) | | 742 (52.4) | |  |
| In the balcony | 687 (31.0) | | 468 (33.1) | | 0.002 |
| **Had access to the street** | |  | |  | |
| No | 1,651 (76.8) | | 1,021 (72.2) | |  |
| Yes | 498 (23.2) | | 394 (27.8) | | 0.002 |
| **Shampoo to flea and tick** | |  | |  | |
| No | 1,275 (59.3) | | 875 (61.9) | |  |
| Yes | 874 (40.7) | | 538 (38.1) | | 0.121 |
