# Supplementary material for: Effectiveness of deltamethrin-impregnated dog collars on the incidence of canine infection by Leishmania infantum: A large scale intervention study in an endemic area in Brazil
Source: PLoS One. 2018 Dec 10;13(12):e0208613. doi: 10.1371/journal.pone.0208613 (PMC6287856; doi:10.1371/journal.pone.0208613)
Supplement: S4 Table — (DOCX) [file pone.0208613.s007.docx]

**S4 Table:** Univariate analysis of the characteristics of the dogs.

| **Variable** | **Intention-to-treat**  **HR (95% CI)** | ***P*** | **Per-protocol HR (95% CI)** | ***P*** |
| --- | --- | --- | --- | --- |
| **Sex** |  |  |  |  |
| Male |  |  |  |  |
| Female | 0.8 (0.6−1.0) | 0.105 | 0.8 (0.6−1.0) | 0.098 |
| **Size** |  |  |  |  |
| Small |  |  |  |  |
| Medium | 1.6 (1.2−2.0) | 0.001 | 1.5 (1.0−2.0) | 0.014 |
| Big | 1.5 (1.0−2.3) | 0.049 | 1.3 (0.8−2.1) | 0.304 |
| **Fur length** |  |  |  |  |
| Long |  |  |  |  |
| Short | 1.6 (1.3−2.2) | 0.001 | 1.6 (1.2−2.2) | 0.004 |
| **Veterinary check-ups** |  |  |  |  |
| Yes |  |  |  |  |
| No | 0.5 (0.3−0.7) | 0.001 | 0.5 (0.3−0.8) | 0.002 |
| **Place where dogs lived and rested** |  |  |  |  |
| Inside the house |  |  |  |  |
| In the backyard | 4.8 (2.9−8.0) | 0.001 | 5.6 (3.0−10.7) | 0.000 |
| In the balcony | 2.2 (1.3−3.9) | 0.004 | 2.5 (1.2−4.9) | 0.010 |
| **Sleeping place** |  |  |  |  |
| Inside the house |  |  |  |  |
| In the backyard | 4.4 (2.7−7.2) | 0.001 | 4.7 (2.6−8.4) | 0.001 |
| In the balcony | 2.0 (1.2−3.5) | 0.011 | 2.1 (1.1−4.0) | 0.020 |
| **Had access to the street** |  |  |  |  |
| No |  |  |  |  |
| Yes | 1.7 (1.3−2.2) | 0.001 | 1.6 (1.1−2.1) | 0.005 |
| **Shampoo to flea and tick** |  |  |  |  |
| No |  |  |  |  |
| Yes | 0.5 (0.4−0.6) | 0.001 | 0.5 (0.4−0.7) | 0.001 |
