# Supplementary material for: Effectiveness of deltamethrin-impregnated dog collars on the incidence of canine infection by Leishmania infantum: A large scale intervention study in an endemic area in Brazil
Source: PLoS One. 2018 Dec 10;13(12):e0208613. doi: 10.1371/journal.pone.0208613 (PMC6287856; doi:10.1371/journal.pone.0208613)
Supplement: S5 Table — (DOCX) [file pone.0208613.s008.docx]

**S5 Table:** Univariate analysis of the characteristics of owners, domicile and peridomicile of the dogs.

| **Variable** | **Intention-to-treat**  **HR (95% CI)** | ***P*** | **Per-protocol HR (95% CI)** | ***P*** |
| --- | --- | --- | --- | --- |
| **Schooling** |  |  |  |  |
| Illiterate |  |  |  |  |
| Primary school | 0.7 (0.5−0.9) | 0.011 | 0.5 (0.5−0.8) | 0.001 |
| Secondary School/University | 0.8 (0.6−1.1) | 0.107 | 0.8 (0.5−1.1) | 0.104 |
| **Home** |  |  |  |  |
| Own or financed |  |  |  |  |
| Rented | 0.7 (0.5−1.1) | 0.117 | 0.6 (0.4−1.1) | 0.103 |
| **Socioeconomic class*** |  |  |  |  |
| A2/ B1 |  |  |  |  |
| B2 | 0.9 (0.4−1.9) | 0.806 | 1.1 (0.5−2.7) | 0.828 |
| C1 | 1.3 (0.7−2.6) | 0.408 | 1.6 (0.7−3.7) | 0.257 |
| C2/D/E | 1.7 (0.9−3.4) | 0.113 | 1.9 (0.8−4.4) | 0.114 |
| **Previous case of CVL in the household** | |  |  |  |
| No |  |  |  |  |
| Yes | 1.4 (1.1−1.8) | 0.005 | 1.4 (1.0−1.8) | 0.033 |
| **Backyard** |  |  |  |  |
| No |  |  |  |  |
| Yes | 2.2 (1.6−3.0) | 0.001 | 2.1 (1.5−3.0) | 0.001 |
| **Backyard features** |  |  |  |  |
| Cement |  |  |  |  |
| Ground | 1.7 (1.2−2.5) | 0.005 | 2.1 (1.4−3.1) | 0.001 |
| Cement and ground | 1.8 (1.3−2.5) | 0.001 | 2.0 (1.4−3.0) | 0.001 |
| **Plant bed in the backyard** |  |  |  |  |
| No |  |  |  |  |
| Not applicable | 0.4 (0.3−0.6) | 0.001 | 0.5 (0.3−0.7) | 0.001 |
| Yes | 1.0 (0.8−1.3) | 0.908 | 0.9 (0.7−1.3) | 0.755 |
| **Banana tree in the backyard** |  |  |  |  |
| No |  |  |  |  |
| Not applicable | 0.5 (0.3−0.6) | 0.001 | 0.5 (0.3−0.7) | 0.001 |
| Yes | 1.4 (0.9−1.9) | 0.062 | 1.3 (0.9−2.0) | 0.146 |
| **Garbage in the backyard** |  |  |  |  |
| No |  |  |  |  |
| Not applicable | 0.5 (0.3−0.7) | 0.001 | 0.5 (0.3−0.8) | 0.001 |
| Yes | 1.2 (0.9−1.6) | 0.118 | 1.3 (0.9−1.9) | 0.102 |
| **Dry leaves in the backyard** |  |  |  |  |
| No |  |  |  |  |
| Not applicable | 0.5 (0.4−0.7) | 0.001 | 0.5 (0.3−0.8) | 0.001 |
| Yes | 1.4 (0.9−1.6) | 0.020 | 1.2 (0.9−1.7) | 0.179 |
| **Trees in the backyard** |  |  |  |  |
| No |  |  |  |  |
| Not applicable | 0.6 (0.4−0.8) | 0.005 | 0.6 (0.4−0.9) | 0.039 |
| Yes | 1.5 (1.1−1.9) | 0.010 | 1.5 (1.1−2.1) | 0.020 |
| **Manure in the backyard** |  |  |  |  |
| No |  |  |  |  |
| Not applicable | 0.4 (0.3−0.6) | 0.001 | 0.5 (0.3−0.7) | 0.001 |
| Yes | 1.1 (0.6−1.7) | 0.828 | 1.0 (0.6−1.7) | 0.972 |

***** A2/ B1= class that receives more than 10 minimum wages.

B2 = class that receives between 6 and 10 minimum wages.

C1 = class that receives between 4 and 6 minimum wages.

C2/D/E = class that receives less than 4 minimum wages.

Brazilian monthly minimum wage = R$788.00 / ~ U$329.
